# Latent class analysis of the diagnostic characteristics of PCR and conventional bacteriological culture in diagnosing intramammary infections caused by *Staphylococcus aureus* in dairy cows at dry off

**DOI:** 10.1186/1751-0147-54-65

**Published:** 2012-11-20

**Authors:** Sara Ellinor Cederlöf, Nils Toft, Bent Aalbaek, Ilka Christine Klaas

**Affiliations:** 1 Hushållningssällskapet Västernorrland, Trädgårdsgatan 7, Härnösand, Sweden; 2 Department of Large Animal Science, University of Copenhagen, Grønnegårdsvej, Frederiksberg C, Denmark; 3 Department of Veterinary Disease Biology, University of Copenhagen, Ridebanevej, Frederiksberg C, Denmark

**Keywords:** Staphylococcus aureus, PCR, Latent class analysis, Sensitivity, Specificity, Mastitis, Bacteriological culture

## Abstract

**Background:**

*Staphylococcus aureus* is one of the most common causes of intramammary infections in dairy cows at dry off. Reliable identification is important for disease management on herd level and for antimicrobial treatment of infected animals. Our objective was to evaluate the test characteristics of PathoProof ™ Mastitis PCR Assay and bacteriological culture (BC) in diagnosing bovine intramammary infections caused by *S. aureus* at dry off at different PCR cycle threshold (Ct)-value cut-offs.

**Methods:**

Sterile quarter samples and non-sterile composite samples from 140 animals in seven herds were collected in connection with the dairy herd improvement (DHI) milk recording. All quarter samples were analyzed using BC whereas all composite samples were analyzed with PathoProof ™ Mastitis PCR Assay. Latent class analysis was used to estimate test properties for PCR and BC in the absence of a perfect reference test. The population was divided into two geographically divided subpopulations and the Hui-Walter 2-test 2-populations model applied to estimate Se, Sp for the two tests, and prevalence for the two subpopulations.

**Results:**

The Se for PCR increased with increasing Ct-value cut-off, accompanied by a small decrease in Sp. For BC the Se decreased and Sp increased with increasing Ct-value cut-off. Most optimal test estimates for the real-time PCR assay were at a Ct-value cut-off of 37; 0.93 [95% posterior probability interval (PPI) 0.60-0.99] for Se and 0.95 [95% PPI 0.95-0.99] for Sp. At the same Ct-value cut-off, Se and Sp for BC were 0.83 [95% PPI 0.66-0.99] and 0.97 [95% PPI 0.91-0.99] respectively. Depending on the chosen PCR Ct-value cut-off, the prevalence in the subpopulations varied; the prevalence increased with increasing PCR Ct-value cut-offs.

**Conclusion:**

Neither BC nor real-time PCR is a perfect test in detecting IMI in dairy cows at dry off. The changes in sensitivity and prevalence at different Ct-value cut-offs for both PCR and BC may indicate a change in the underlying disease definition. At low PCR Ct-value cut-offs the underlying disease definition may be a truly/heavily infected cow, whereas at higher PCR Ct-value cut-offs the disease definition may be a *S. aureus* positive cow.

## Background

*Staphylococcus aureus* is one of the most important causes of intramammary infections (IMI) in dairy cows at dry off [1]. The majority of cases are subclinical. A recent study reports that *S. aureus* was the second most commonly isolated pathogen in samples collected pre dry off [2] and a prevalence of 8.2% has been reported for quarter level samples from dairy cows at dry off [3]. Selective dry cow treatment is receiving more attention since there is an increasing focus on proper use of antibiotics. Blanket dry cow treatment is prohibited in Denmark and selective dry cow treatments are only allowed after a microbiological diagnosis [4]. Reliable identification of cows with *S. aureus* IMI is important for disease management on herd level, for selecting cows for dry cow treatment and in general for appropriate antimicrobial treatment of infected animals. Bacteriological culture (BC) is considered the standard in diagnosing bovine IMI [5], but approximately 39% of milk samples from subclinical cases of IMI showed no growth on BC [6]. Studies have shown ranges of sensitivity (Se) between 0.75 to 0.93 for single samples analyzed with BC [7,8]. *S. aureus* are known to be shed in a cyclic pattern from infected quarters [7] and repeated sampling over time can be necessary in order to increase the Se [7,9]. However, a recent study showed that culture of a single milk sample resulted in the highest Se and specificity (Sp), 0.904 and 0.998 respectively for *S. aureus,* when IMI was defined as one or more colonies in pure or mixed culture [10].

The use of molecular methods to detect pathogens has increased over the last years. A real-time polymerase chain reaction (PCR) based reagent kit available in Denmark, PathoProof ™ Mastitis PCR Assay (Finnzymes Oy, Espoo, Finland) can target 11 different IMI causing bacterial species and groups and analyze for penicillin resistance among *Staphylococci*[11]. Cycle threshold (Ct) -value is a key variable when evaluating real-time PCR results. The Ct-value represents the number of cycles required to reach a particular threshold fluorescence signal level. The fewer cycles required to reach the threshold level, the greater amount of DNA in the sample [12]. An extensive review regarding the choice of Ct-value cut-off in real-time PCR assays, given from both an epidemiological and an analytical point of view is written by Caraguel et al. 2011 [13]. PathoProof ™ Mastitis PCR Assay gives a semi-quantitative result (+, ++, +++) where + equals a Ct-value cut-off between 30 and 37, ++ 24–30 and +++ < 24 respectively for *S. aureus*[14].

Several studies suggest that assays based on PCR could serve as a useful tool in diagnosing IMI pathogens [15-17]. Arguments in favor of PCR include the short analysis time compared to BC, the ability to detect growth-inhibited and dead bacteria and PCR’s high analytical sensitivity. Evidence indicates that PCR may produce a diagnosis in cases of clinical mastitis or subclinical mastitis where BC turned out negative [11,18]. Others have shown that the Se and Sp for the PCR assay for *S. aureus* and all major pathogens to range between 0.86 to 0.94 and 0.91 to 0.95 respectively [19,20].

In Denmark, PCR analysis has been allowed to substitute BC for microbiological diagnosis prior to selective dry cow treatment since July 2010. In 2012, PCR testing of cows after day 200 in lactation was reported from 767 Danish dairy farms [21]. Farmers can order PCR tests on cow-level samples from routine dairy herd improvement (DHI) milk recordings, which are analyzed using PathoProof ™ Mastitis PCR assay [4,22]. Although Denmark already utilizes the PCR assay in routine mastitis testing, relatively few published studies evaluate its accuracy under field circumstances for clinical cases of mastitis [11,18] and to our knowledge no study evaluates its accuracy for IMI based on cow-or quarter level samples.

Regarding diagnosis of IMI, there is no perfect gold standard [23]. If using a non-perfect test as reference standard when evaluating new test methods, new methods will be punished for diagnosing correctly if the non-perfect test does not. This will result in an underestimation of Se and Sp [24]. Using latent class analysis (LCA), it is possible to estimate Se and Sp without having a gold standard. The disease status exists but is not known; it is latent [25,26].

The objective of this study was to evaluate the Se and Sp of PathoProof ™ Mastitis PCR Assay and BC in diagnosing bovine IMI caused by *S. aureus* at dry off with LCA. Specifically we tested the effect of using different PCR Ct-value cut-offs on the Se and Sp of both tests.

## Methods

### Herds and animals

The source population for this study was all Danish dairy herds using PCR to diagnose IMI in their cows at dry off, which is the main reason for farmers to order PCR tests. A convenience sample of seven Danish herds was included in this study. The herds were selected based on geographic location and willingness to participate during two weeks of March 2011. The herds had to expect at least ten pre dry off cows for PCR at their next DHI milk recording. The cows selected for PCR sampling were dried off within a week after the DHI milk recording. The farmers selected cows for PCR test mainly based on previous somatic cell counts. The farmers ordered PCR sampling for the selected cows for the subsequent routine DHI recording in the Danish Cattle Database where the results of the PCR tests are registered.

### Samples for PCR

Milk samples submitted for the PathoProof ™ Mastitis PCR Assay were collected automatically at cow-level during DHI milk recording under non-sterile conditions in sample tubes containing bronopol to restrict bacterial growth during the routine milk recording. In herds with milking parlour, the samples for PCR were metered by Tru-Test electronic milk meter. Samples were handled either by the manager/staff of the farms or a technician. In automatic milking system (AMS) herds, composite samples were taken by the Lely Shuttle milk sampling unit. Milk samples from the DHI milk recording were transported by a technician to Eurofins│Steins in Holstebro, Denmark. Samples taken during the morning milking arrived at the laboratory the same day, samples taken during evening milking arrived the following day.

### Samples for bacteriological culture

Within 24 hours after PCR sampling, the first author collected sterile quarter foremilk samples for BC from the same cows. Following disinfection of the teat ends using cotton swabs soaked in 70% alcohol and let to dry, aseptic samples (app. 10 mL) were collected in sterile plastic tubes. The first few mL of milk was discarded and latex gloves were worn during the sampling. In the five herds with milking parlour, pre-milking quarter samples were collected. In the two AMS herds, quarter milk samples were collected in the separation area at the exit of the robotic unit. Samples for bacterial culture were frozen at – 20°C after sampling and stored until further analysis.

### Bacterial culture

The milk samples where thawed at room temperature, i.e. 20-25°C, before plating. Volumes of 0.01 mL of milk samples were plated on blood agar, prepared from Oxoid blood agar base (Oxoid, Basingstoke, Hampshire, England) supplemented with 5% sterile bovine blood. The cultures were incubated aerobically at 37°C and read after 1 and 2 day’s incubation. Representative colonies were sub-cultured. Colony morphology, hemolysis, cell shape, Gram stain ability and catalase reaction were recorded. Cultures having intermediate sized, smooth, white or yellow, hemolytic colonies consisting of catalase positive, Gram positive cocci, were considered as staphylococci [27,28]. Such isolates were subjected to coagulase testing, using equine plasma [29]. When ≥ 1 CFU of coagulase positive staphylococci were demonstrated in at least one mammary quarter of a cow, it was included as positive. Samples containing more than three different colony types were considered as contaminated and the cow was excluded from the study.

### Real-time PCR assay

A commercial real-time PCR test kit (PathoProof™ Mastitis PCR Assay, Finnzymes Oy, Espoo, Finland) was used for analysis of the composite milk samples collected at the milk recordings. The milk samples were kept refrigerated during transportation to the laboratory and until analyzed with PCR. The PCR test was performed by Eurofins│Steins in Holstebro, Denmark. The laboratory follows standard procedures for the PCR assay described elsewhere [30].

### Statistical analysis

To estimate Se and Sp for the PathoProof ™ Mastitis PCR Assay and BC, a Bayesian latent class analysis was used. The analysis was based on the Hui-Walter paradigm: (i) the tests are conditionally independent (ii) the population is divided into two subpopulations with different disease prevalence and (iii) sensitivity and specificity of the tests is the same in all populations [25]. The population was divided into two subpopulations based on geographical location, with herds located on Jutland/Fyn and herds located on Zealand. The Hui-Walter 2-test 2-populations model was used to estimate Se, Sp and prevalence for the two tests, PCR and BC, and for the two subpopulations. The Bayesian version of a latent class analysis was conducted in OpenBUGS version 3.1.2 [31]. This software uses a Markov Chain Monte Carlo (MCMC) sampling algorithm to obtain a Monte Carlo sample from the posterior distribution. The first 10.000 samples were discarded as burn-in to allow convergence. The following 50.000 iterations were used for posterior inference. Uninformative priors Beta (1, 1) were used for all parameters. The MCMC chain was after initial burn-in, assessed by visual inspection of time-series plots of variables as well as Gelman-Rubin diagnostic plots, to assess whether convergence had appeared [32]. Posterior inference presented medians and corresponding 95% posterior probability intervals (PPI, which can be seen as the Bayesian analogue of a confidence interval) for the prevalence in the two subpopulations and Se and Sp for the two tests, PCR and BC. As explained in Toft et al. 2007 [24], Bayesian posterior probabilities (POPR) were calculated in order to decide in favor of or against one of the two tests at the pre-defined PCR Ct-value cut-off for positive samples; 37. To investigate the effect of low and moderate concentration of DNA in the samples on the test characteristics of both tests, latent class analysis was carried out at PCR Ct-value cut-offs ≤ 32, ≤ 34, ≤ 37 and ≤ 39.

## Results

### Descriptive statistics

During editing 17 animals were excluded from analysis because of the following reasons: For 10 animals, one or several quarter samples for BC could not be reliably identified, four animals had missing results from the PCR assay and three animals had one or several quarter samples that were classified as contaminated i.e. samples with more than three different bacterial colonies. After editing complete samples from 140 Danish Holstein-Friesian and Jersey dairy cows from seven different herds were eligible for analysis. One of the herds was tested at DHI milk recording in the start of early April 2011 and from one included herd, only six cows were at dry off and selected for PCR sampling at the DHI milk recording.

The cows included in this study had a SCC on the test day between 8.000 – 5.340.000 cells/mL, with an average of 483.000 (CI 95% 343.000; 623.000). The cows produced on the test day between 8.5 – 34 kg of milk with an average of 23 kg (CI 95% 22; 24). Parity ranged from one to 13, with an average of 2.6 (CI 95% 2.3; 2.9).

Cross-classified results of the PCR and BC for selected Ct-value cut-offs are shown in Table 1 where the results are divided into the two subpopulations, Jutland/Fyn and Zealand.

**Table 1:** **The total number of cows found positive for *S. aureus *in two geographically divided subpopulations by PCR and bacteriological culture (BC), as a function of the Ct-value cut-off for PCR**

**Ct-value cut-offPCR**	**BC pos / PCR pos**	**BC pos / PCR neg**	**BC neg / PCR pos**	**BC neg / PCR neg**
Jutland/Fyn (n = 80)				
≤ 32	10	15	0	55
≤ 34	17	8	4	51
≤ 37	23	2	8	47
≤ 39	23	2	11	44
Zealand (n = 60)				
≤ 32	8	6	1	45
≤ 34	11	3	2	44
≤ 37	12	2	3	43
≤ 39	12	2	4	42

### Test characteristics for PCR and bacteriological culture

Latent class estimates of Se and Sp for BC and PCR are presented in Table 2 at different PCR Ct-value cut-offs. The Se for PCR increased as expected when the Ct-value cut-off increased, albeit with a corresponding (but smaller) decrease in Sp. For BC the Se decreased and Sp increased with increasing PCR Ct-value cut-off. Similarly, Table 3 gives the prevalence in the two populations where an increased prevalence is also observed with increasing PCR Ct-value cut-off. When calculating the POPR for the Sp at PCR Ct-value cut-off 37 against Sp of BC, it was found to be 0.305, i.e. in only 30% of the cases will the PCR assay be the most specific method. This illustrates that there were no statistical evidence that one of the two tests should be more specific.

**Table 2:** **Estimate and 95 % posterior probability interval (PPI) of sensitivity (Se) and specificity (Sp) of PCR and bacteriological culture (BC) at PCR Ct-value cut-off 32, 34, 37 and 39, as tests for diagnosing intramammary infections by *S. aureus***

	**PCR**				**Bacteriological culture**			
**Ct-value cut-off PCR**	**Se**	**95 % PPI**	**Sp**	**95 % PPI**	**Se**	**95 % PPI**	**Sp**	**95 % PPI**
≤ 32	0.61	0.36 ; 0.97	0.99	0.95 ; 0.99	0.94	0.76 ; 0.99	0.90	0.79 ; 0.99
≤ 34	0.81	0.61 ; 0.99	0.96	0.90 ; 0.99	0.88	0.70 ; 0.99	0.94	0.86 ; 0.99
≤ 37	0.93	0.80 ; 0.99	0.95	0.85 ; 0.99	0.83	0.66 ; 0.99	0.97	0.91 ; 0.99
≤ 39	0.93	0.80 ; 0.99	0.93	0.82 ; 0.99	0.78	0.60 ; 0.98	0.97	0.91 ; 0.99

**Table 3:** Estimates and 95 % posterior probability interval (PPI) of prevalence in the two populations; Jutland/Fyn and Zealand as a function of the Ct-value cut-off for PCR

	**Prevalence**			
**Ct-value cut-off PCR**	**Jutland/Fyn**	**95 % PPI**	**Zealand**	**95 % PPI**
≤ 32	0.23	0.10 ; 0.39	0.20	0.09 ; 0.34
≤ 34	0.30	0.18 ; 0.44	0.23	0.13 ; 0.36
≤ 37	0.37	0.24 ; 0.50	0.25	0.14 ; 0.38
≤ 39	0.40	0.26 ; 0.53	0.26	0.14 ; 0.39

## Discussion

This study estimated the test characteristics of a commercially available PCR assay, PathoProof ™ Mastitis PCR Assay, and BC to identify IMI with *S. aureus* in dairy cows at dry off. The study was done using LCA to avoid the assumption of an available perfect reference test. The findings were ambiguous. PCR had lower Se and higher Sp than BC for low PCR Ct-value cut-offs, but it was reversed for higher PCR Ct-value cut-offs. Surprisingly, Se and Sp of BC were affected by the choice of the PCR Ct-value cut-off. Increasing prevalence at increasing PCR Ct-value cut offs support the assumption of a quantitative relationship between Ct-values and infection load in the sample [12].

A PCR assay detects DNA from both viable and non-viable bacteria while BC is only capable of detecting viable bacteria [11]. In cases where the infection is cured, either due to treatment or self-cure, bacterial DNA can still be present in the udder and the cow can be falsely diagnosed as positive by the PCR assay [33]. When increasing the PCR Ct-value cut-off, the Se of BC decreases, indicating that the bacteria detected by PCR could be non-viable, in amounts lower than the detection threshold of the BC or derived from other sources than the milk. When using a high PCR Ct-value cut-off, more animals may be false positive by PCR due to different reasons for example non-viable bacteria in low concentrations. Since *S. aureus* commonly inhabits cow skin [34], the bacteria could furthermore be a result of contamination, teat canal infections or teat skin infections. Carry-over in the milking system could also be a possibility [35]. The results also indicate that the disease definition is changing depending on the PCR Ct-value cut-off. The effect is shown in the prevalence estimates where a PCR Ct-value cut-off of 32 results in a disease prevalence of 23% in subpopulation Jutland/Fyn. When the PCR Ct-value cut-off is increased to 37, the disease prevalence has increased to 37% in the same population. One could argue that a PCR Ct-value cut-off at 32 indicates the presence of a high amount of bacteria in the milk, which most likely are associated with an active IMI. When the amount of bacteria in the milk is high, we increase the probability that viable bacteria are present, which can be detected by BC. When increasing the PCR Ct-value cut-off, the disease definition is apparently changing, reflected by an increasing prevalence. Higher PCR Ct-values may indicate that the amount of bacteria is below the detection threshold of BC, that the bacteria present are non-viable and/or that the bacteria derive from other sources than the infected mammary gland. Under the assumption that the disease definition changes dependent on the chosen PCR Ct-value cut-off, changes in test estimates for both tests at different PCR Ct-value cut-off for the real-time PCR may be a logical consequence.

In this study however, both BC and the real-time PCR assay had relatively high Se and Sp, depending on the PCR Ct-value cut-off, indicating that both tests can serve as useful tools in diagnosing IMI caused by *S. aureus.* Friendship et al., 2010 [19] evaluated the PathoProof ™ Mastitis PCR Assay in detecting *S. aureus*. All cows in the study originated from herds with a known history of intramammary infections due to *S. aureus*. The study found a Se for the PCR Assay of 0.94 and Sp of 0.95 when using BC as gold standard. Koskinen et al. 2010 [20], found the Se and Sp for the PathoProof ™ Mastitis PCR Assay to be 0.862 and 0.916 respectively when evaluated for all pathogens [20]. These Se and Sp are similar to those found in this study, where Se and Sp for the PCR assay were estimated to 0.93 and 0.95 respectively at PCR Ct-value cut-off 37. The later article argues that the use of BC as reference standard most likely results in an underestimation of test estimates for the PCR due to inaccuracy of the reference test. Our study can support this assumption and support the use of LCA in situations where no perfect reference test is available.

All milk samples used for BC in this study were frozen prior to analysis. Freezing has been shown to both increase the likelihood for obtaining *S. aureus* in the milk sample [36-38] and decrease or to show no effect in detection rates [39-41]. Evaluating the effect of freezing on BC results was beyond the scope of our study. Thus, we cannot exclude that freezing might have affected the results.

PCR is shown to be able to detect mastitis bacteria in samples that yielded no growth in bacteriological culture [18], which is an advantage in cases where the aetiology of the mastitis is of special interest. Another advantage is the speed of the real-time PCR analysis compared to BC; there is a possibility for the same-day results.

When interpreting the Ct-value given by the PCR assay, one should consider the type of sample and the sampling conditions. The samples for PCR were in this study collected under non-sterile conditions and metered by the milking equipment. In these samples there is a possibility for contamination from the previous cows and other sources than the milk. Pre-milking practices in the herd and condition of the milking equipment may have an influence on the obtained Ct-values. However, differences between the included herds made the estimation of these factors not possible. The results given in form of a Ct-value should furthermore be interpreted with all other available information for the cow, such as history, clinical signs and SCC in the milk. This should however also be the practice with results from BC.

### Model assumptions

An implied assumption of the LCA is that the two tests are conditionally independent given disease status. Given that there is no culturing involved in the PCR procedure, this assumption is justified, however, only when accepting the underlying disease definitions discussed above.

In this study, the population was divided based on geographical location, Jutland/Fyn and Zealand. The difference in prevalence found here was not large, but as the herds were selected to present problems, we could not expect large differences. In Toft et al. 2005 [26], the importance of the difference in true prevalence was examined. Although smaller differences might infer some bias, it is more important that the assumption about constant Se and Sp across populations is justified.

Both farms using AMS and conventional milking systems were included in the study. A total of 25 cows originated from AMS herds. A study by Lovendahl and Bjerring [35], indicates that carryover problems are not negligible in the AMS system compared to conventional systems. It could be assumed that a higher proportion of cows could be classified as false positive due to carryover. Excluding the 25 cows originating from AMS herds did not result in any notable differences on the estimates of test accuracy.

## Conclusion

Results in this study indicate that neither BC nor real-time PCR is a perfect test in detecting IMI in dairy cows at dry off. The changes in sensitivity and prevalence at different Ct-value cut-offs for both PCR and BC may indicate a change in the underlying disease definition. At low PCR Ct-value cut-offs the underlying existing, but latent disease definition may be a truly/heavily infected cow, whereas at higher PCR Ct-value cut-offs the disease definition may be a *S. aureus* positive cow.

## Competing interests

The authors declare that they have no competing interests.

## Authors’ contributions

The study was designed by SEC and ICK. SEC performed the collection of samples, the laboratory work and the analysis of results was done by all authors. NT and SEC performed all statistical calculations. BA supervised the bacteriological culturing. All authors revised, read and approved the final manuscript.
